# Prognostic utility of plasma lactate measured between 24 and 48 h after initiation of early goal-directed therapy in the management of sepsis, severe sepsis, and septic shock

**DOI:** 10.1186/s40560-016-0142-7

**Published:** 2016-02-12

**Authors:** Jason Chertoff, Michael Chisum, Lauren Simmons, Brent King, Michael Walker, Jorge Lascano

**Affiliations:** Department of Medicine, College of Medicine, University of Florida, 1600 SW Archer Road, Gainesville, FL 32608 USA; Division of Pulmonary Critical Care, University of Florida, Gainesville, FL USA

**Keywords:** Lactate, Clearance, Sepsis, Early sepsis, Late sepsis, Resuscitation, Mortality, Vasopressor, Microvascular, Shock

## Abstract

**Background:**

Based on the proven efficacy of lactate in predicting mortality and morbidity in sepsis when measured early in the resuscitative protocol, our group hypothesized that this utility extends later in the course of care. This study sought to investigate the prognostic potential of plasma lactate clearance measured 24–48 h after the initiation of treatment for nonsurgical patients with sepsis, severe sepsis, and septic shock.

**Methods:**

Plasma lactate values, measured 24–48 h after the initiation of treatment, were collected in nonsurgical septic, severe septic, and septic shock patients. The primary outcome was 30-day mortality, while secondary outcomes included requirements for vasopressors and boluses of intravenous fluids. Analysis of these three outcomes was performed while controlling for clinical severity as measured by Sequential Organ Failure Assessment (SOFA), renal dysfunction, and hepatic dysfunction. Lactate clearance was defined as the percent change in plasma lactate levels measured after 24–48 h of treatment from the plasma lactate level at initial presentation.

**Results:**

Two hundred twenty-nine nonsurgical patients were divided into two groups, clearers (above median lactate clearance [31.6 %]) and nonclearers (below median lactate clearance [31.6 %]). The adjusted odds ratio of mortality in clearers compared to nonclearers was 0.39 (CI 0.20–0.76) (p = 0.006). For vasopressor requirement, the adjusted odds ratio was 0.41 (CI 0.21–0.79) in clearers compared to nonclearers (p = 0.008). For intravenous fluid bolus requirement, the adjusted odds ratio was 0.81 (CI 0.48–1.39) in clearers compared to nonclearers (p = 0.45).

**Conclusions:**

Lower plasma lactate clearance 24–48 h after the initiation of treatment is associated with higher 30-day mortality and requirements for vasopressors in nonsurgical septic patients and may be a useful noninvasive measurement for guiding late-sepsis treatment. Further investigation looking at mechanisms and therapeutic targets to improve lactate clearance in late sepsis may improve patient mortality and outcomes.

## Background

The rate of sepsis-related intensive care unit (ICU) admissions has increased steadily over the last decade, with hospitalizations for sepsis doubling and close to one million Americans affected annually [1–3]. Nearly a decade since the landmark article, “Surviving Sepsis Campaign Guidelines for Management of Severe Sepsis and Septic Shock”, sepsis remains a hotbed of research, and new diagnostic and resuscitative interventions are continually under development and evaluation. One such effort that has received focus is the role of lactate monitoring [1, 3–5].

In their 2004 and 2008 sepsis guidelines, Dellinger et al. recommended measurement of lactate on initial presentation [1, 6–8]. In addition, many clinicians and researchers have attempted to capitalize on the test’s theoretical diagnostic and predictive value by including additional measurements during the resuscitation process [2, 9–12]. For example, it was shown that lactate clearance greater than 10 % from initial measurement during the first 2 to 6 h of resuscitation predicted survival from septic shock and that protocols targeting lactate clearance of at least 10 % produced similar short-term survival rates to protocols using central venous oxygen saturation (ScVO2) monitoring [2, 3, 9, 11].

In addition to lactate clearance’s utility early in the resuscitation process, there have been some investigations of lactate’s predictive value at later time periods [13–17]. In a 137 Surgical Intensive Care Unit (SICU)-patient study, Husain et al. showed elevated initial and 24-h lactate levels to be significant predictors of mortality, with mortality ranging from 10 to 67 % depending on whether lactate levels normalized or failed to normalize after 24 h, respectively [18]. In another study investigating SICU patients, Bakker et al. showed that lactate clearance measured 24 h after admission was a significant predictor of in-hospital death and that the duration of lactic acidosis was more predictive of mortality than the initial lactate value [13].

These reports suggest that lactate measurements more than 24 h from the initiation of resuscitation have predictive and prognostic utility [19–21]. Motivated by these reports and the lack of explicit guidelines in the medical literature, we sought to investigate the utility and prognostic potential of lactate clearance, measured 24–48 h after the onset of resuscitation. Specifically, our aim was to determine whether lower lactate clearances, measured 24–48 h after the onset of resuscitation, was associated with increased 30-day mortality and requirements for boluses of intravenous fluids and vasopressors in patients treated for sepsis.

## Methods

### Setting

The study was a single-center retrospective cohort study of adult nonsurgical patients with admission diagnoses of sepsis, severe sepsis, and/or septic shock, admitted to UF-Health during 2013, and approved by the Institutional Review Board (IRB) at the University of Florida (UF) College of Medicine, Gainesville, Florida. The patients and their respective diagnoses of sepsis, severe sepsis, and/or septic shock were obtained through database query using ICD coding and confirmed via chart review. This same strategy allowed for exclusion of patients admitted for noninfectious SIRS. All patients were treated at UF-Health, which is a 946-bed quaternary care institution. All patients deemed critically ill were admitted to the UF-Health MICU, which is a 24-bed unit staffed 24 h daily by board-certified pulmonary-critical care physicians, internal medicine residents, and nurses trained and/or board certified in critical care.

### Inclusion and exclusion criteria

**Fig. 1:**
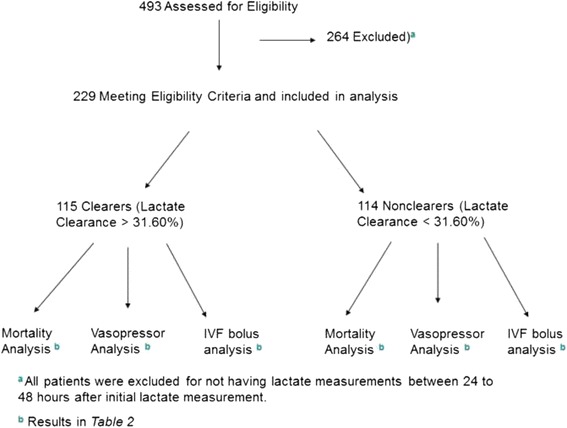
Shows the study flow diagram

Adult patients admitted to UF-Health in Gainesville, Florida, during 2013 with a diagnosis of sepsis, severe sepsis, and/or septic shock were retrospectively chart reviewed. Inclusion criteria included patients with (a) one or more of the aforementioned admission diagnoses in the appropriate time period and (b) an initial lactate measurement followed by one or more lactate measurements taken 24–48 h after resuscitation efforts started. All of these patients had lactate measurements on admission, but only the first lactate measurement taken after 24–48 h was recorded and used for analysis (Fig. 1).

### Patient management

All patients were managed according to our institutional sepsis guidelines including lactate measurement on arrival, fluid resuscitation with evaluation of dynamic measures of fluid response with a goal to achieve a mean arterial blood pressure (MAP) equal or greater than 65 mmHg, vasopressor medications whenever necessary to achieve the goal MAP after fluid resuscitation, infection source control, cultures, and appropriate antibiotic therapy [22]. For all patients, resuscitation efforts were either initiated in the emergency room or on the medical floor. The institutional resuscitation policy for sepsis is identical regardless of the location of initiation (i.e., emergency room, floor). All patients received antibiotic therapy with the initiation of treatment, and although not recorded, the appropriateness of antibiotic therapy did not differ between the two patient groups, which was confirmed via chart review. Depending on respiratory status, patients were treated with oxygen therapy, noninvasive positive pressure ventilation (NIPPV), or intubation with mechanical ventilation.

### Data collection

Patient’s gender, age, admission diagnoses, admission lactate values, and lactate values measured after 24–48 h of resuscitation were recorded. Data collected for Sequential Organ Failure Assessment (SOFA) scores included the worst values obtained within 24 h of resuscitation for the following: PaO2, FiO2, platelet count, Glascow Coma Score (GCS), total bilirubin, creatinine, and either receipt of vasopressors or MAP. Kidney function was recorded as the estimated glomerular filtration rate (EGFR), which was calculated using the Modification of Diet in Renal Disease (MDRD) formula. Liver dysfunction was recorded as the Model for End-Stage Liver Disease (MELD) score, which was calculated using the MELD model. Volume resuscitation with boluses of intravenous fluid (yes/no) and vasopressor therapies (yes/no) administered after 24 h were collected as secondary outcomes.

### Lactate clearance definition

The following formula was used to define lactate clearance: Initial lactate measured at the start of treatment minus lactate measured after 24–48 h, divided by initial lactate (Lactate Clearance = Lactate^Initial^ − Lactate^after 24–48 h^/Lactate^Initial^). A negative value represents an increase in lactate, while a positive value represents a decrease in lactate.

### Clearers vs. nonclearers

**Fig. 2:**
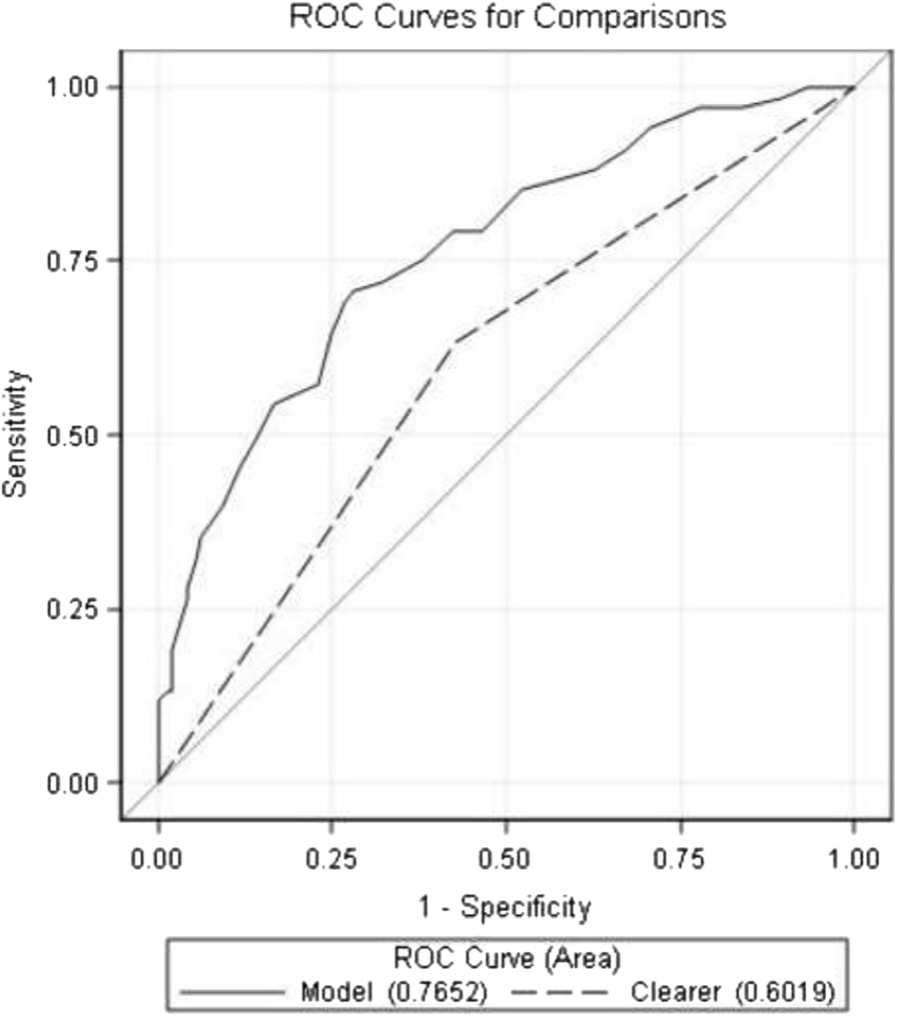
Shows the ROC curve and comparison for the optimal clearance divisor value (25.89 %) and the median clearance value (31.60 %). The figure shows that the two values (25.89 %; 31.60 %) have roughly the same area under the curve (not significantly different using linear discriminant analysis). Since the two values (25.89 and 31.60 %) perform similarly, we were confident in our ability to use the median clearance value, 31.60 %, as our cutoff to divide the two groups of patients (clearers and nonclearers)

Since review of the literature does not clearly define a significant lactate clearance threshold, the median clearance was used to divide the patients into two groups [clearers (patients > median) and nonclearers (patients < median)]. The use of median clearance as a cutoff between clearers and nonclearers was validated using receiver operator curve analyses (Fig. 2). The optimal value was found by linear discriminant analysis using SOFA score as an independent predictor of mortality. The optimal divisor was found to be close to the median value lactate clearance (31.60 %) in the sample of all patients.

### Baseline similarity of clearers vs. nonclearers

The age, diagnosis, EGFR, and MELD score of the patient groups (clearers and nonclearers) were compared to ensure similarity at baseline (Table 1).

**Table 1:** Patient demographics

	Clearers	Nonclearers	*p* (alpha level 0.05)
Age (Avg. years)	60.2	61.3	*p* = 0.250
Sex	61.90 % M/38.1 % F	53.33 % M/46.67 % F	*p* = 0.212
Initial lactate	4.08	2.51	*p* = 0.010
Lactate clearance time (s)	32.0 (SD = 4.93)	31.3 (SD = 4.96)	*p* = 0.782
Diagnosis			
Sepsis	56.36 %	43.64 %	*p* = 0.307
Severe sepsis	55.00 %	45.00 %	*p* = 0.324
Septic shock	45.52 %	54.48 %	*p* = 0.321
Organ function			
Kidney function (EGFR)	47.4	45.9	*p* = 0.531
Liver function (MELD)	15.6	16.6	*p* = 0.363
Total (*N*)	115	114	

### Statistical analysis

The SAS (BASE) version 9.4 and also “R” software programs were used for data analysis. Thirty-day mortality was calculated by dividing the number of deaths within 30 days of ICU admission by the total number of patients analyzed in the study. Statistical significance was defined as *p* < 0.05. Estimates for 30-day mortality comparisons (clearers versus nonclearers) were calculated using odds ratio. Association between clearance groups and outcome was measured with chi-squared testing for independence. In addition, secondary outcomes of intravenous fluid bolus and vasopressor receipt were compared between clearers and nonclearers. Sensitivity analysis for all outcomes was performed using multiple logistic regression analysis with additional covariates in order to control for the effects of confounding related to disease severity and comorbidities. Estimates for each outcome effect, while controlling for SOFA score, EGFR, and MELD, are included in the analysis. Wald tests and 95 % two-sided confidence intervals are presented. The demographic variables of age, sex, and admission diagnosis were analyzed between clearers and nonclearers using the chi-squared test in order to investigate for homogeneity between groups (Table 1).

## Results

### Primary outcome: 30-day mortality

**Fig. 3:**
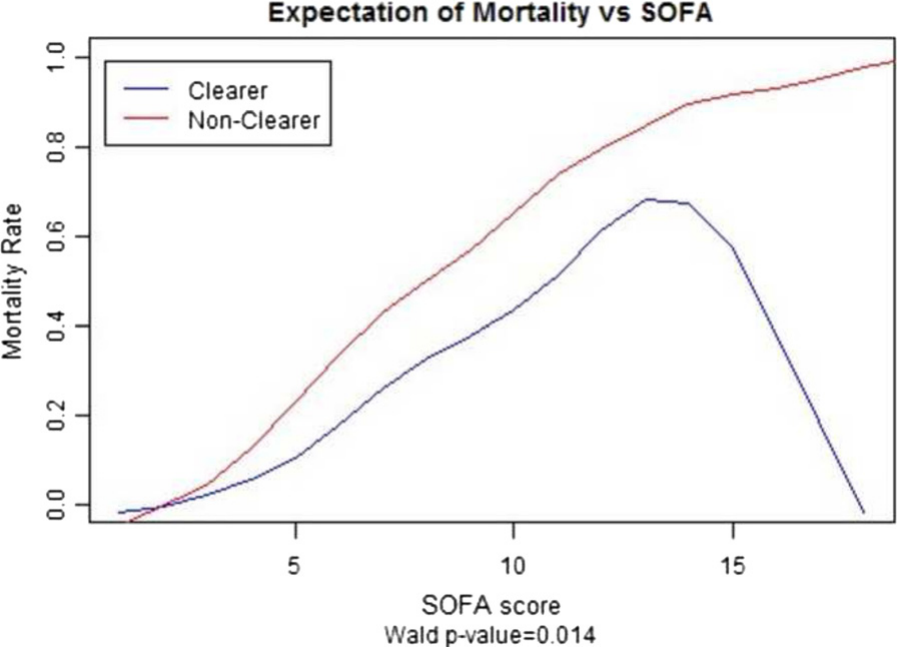
Shows a statistically significant association between mortality and lactate clearance adjusted for SOFA, EGFR, and MELD

**Fig. 4:**
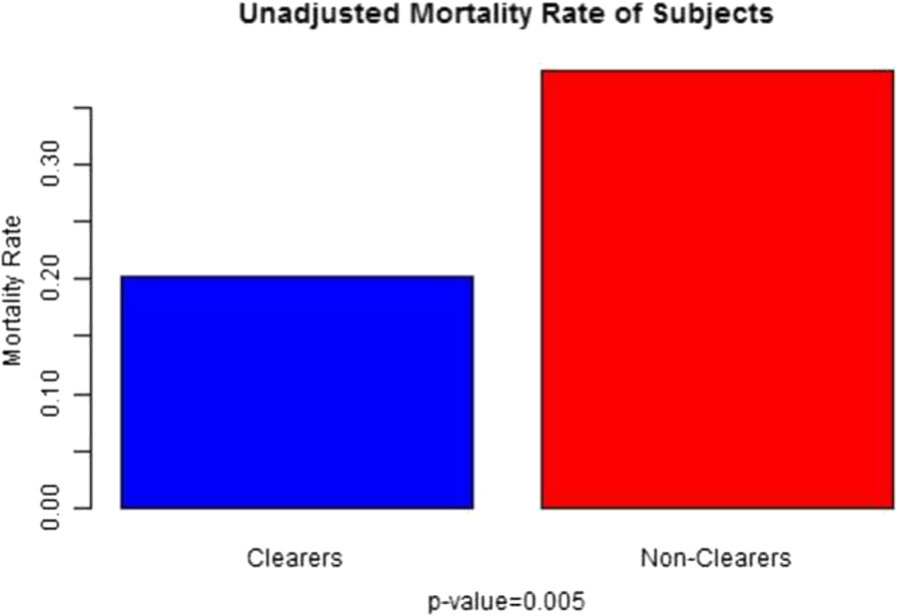
Shows the primary outcome, mortality, with statistically significant differences between the two groups (clearers and nonclearers)

A total of 229 patients, 129 men and 100 women, admitted during 2013 were analyzed (Table 1). With the diagnosis of sepsis, severe sepsis, and septic shock, respectively, 24.0, 17.5, and 58.5 % of the patients were admitted. Patients had a mean baseline SOFA score of 8.26 ± 4.15 and a median lactate clearance of 31.6 % after a mean of 31 h after admission. The 30-day mortality rate was 29.69 %. Before controlling for SOFA, the odds of mortality in clearers compared to nonclearers was 0.44 with 95 % confidence interval (0.24–0.78). A chi-squared test for independence revealed an association between level of clearance and mortality rate (*p* = 0.005) (Table 2). Multiple logistic regression modeling was then performed with lactate clearance, EGFR, MELD, and SOFA score, with the odds ratio and confidence interval being 0.39 (0.20–0.76) (*p* = 0.006), respectively (Figs. 3 and 4).

**Table 2:** Primary and secondary outcomes

	Analysis group	Odds ratio	95 % confidence interval	*p* (alpha level 0.05)
Mortality				
(i) Clearers/nonclearers	Unadjusted OR	0.44	(0.24–0.78)	0.005
(ii) Clearers/nonclearers	OR adjusted for SOFA	0.45	(0.24–0.85)	0.014
(iii) Clearers/nonclearers	OR adjusted SOFA, EGFR, and MELD	0.39	(0.20–0.76)	0.006
Receipt of IV fluids				
(i) Clearers/nonclearers	Unadjusted OR	0.72	(0.43–1.22)	0.22
(ii) Clearers/nonclearers	OR adjusted for SOFA	0.74	(0.44–1.26)	0.27
(iii) Clearers/Nonclearers	OR adjusted SOFA, EGFR, and MELD	0.81	(0.48–1.39)	0.45
Receipt of vasopressors				
(i) Clearers/nonclearers	Unadjusted OR	0.46	(0.27–0.79)	0.005
(ii) Clearers/nonclearers	OR adjusted for SOFA	0.43	(0.23–0.83)	0.012
(iii) Clearers/nonclearers	OR adjusted SOFA, EGFR, and MELD	0.41	(0.21–0.79)	0.008

### Secondary outcomes: need for vasopressors and IVF boluses

#### Vasopressor requirement

**Fig. 5:**
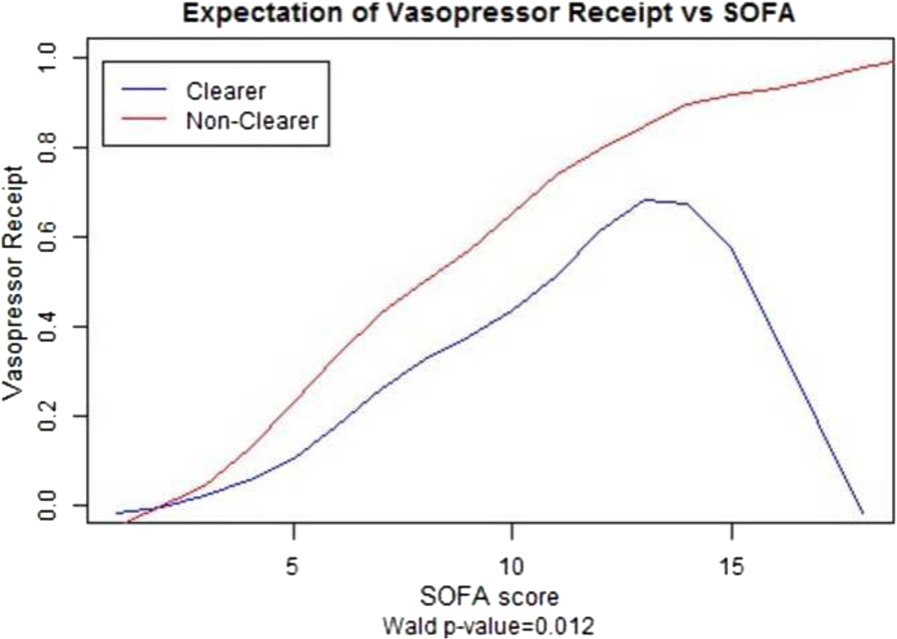
Shows a statistically significant association between vasopressor receipt and lactate clearance adjusted for SOFA, EGFR, and MELD

**Fig. 6:**
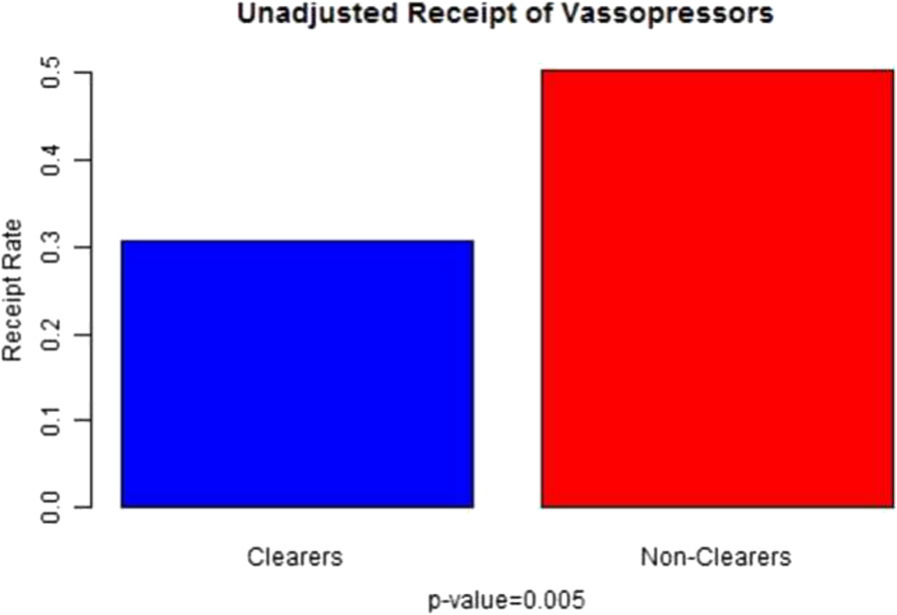
Shows statistically significant differences in vasopressor receipt between the two groups (clearers and nonclearers)

Vasopressor receipt was defined as receiving (yes/no) any dose of dopamine, epinephrine, or norepinephrine 24 h after the initiation of resuscitation efforts. The odds of vasopressor receipt in clearers compared with nonclearers was 0.46 with 95 % confidence interval (0.27–0.79). A test for independence confirmed an association between level of clearance and vasopressor requirement (*p* = 0.005) (Table 2). After adjusting for SOFA score, MELD, and EGFR, multiple logistic regression estimated the odds ratio of requiring vasopressors was 0.41 with 95 % confidence interval (0.21–0.79) (*p* = 0.008) (Figs. 5 and 6).

#### Intravenous fluid (IVF) bolus requirement

**Fig. 7:**
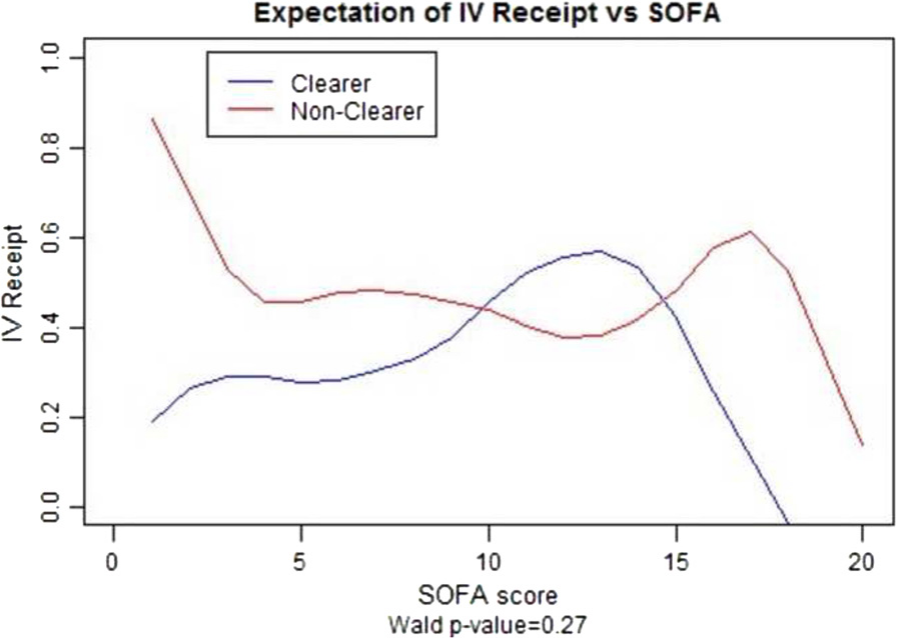
Does not show a statistically significant association between intravenous fluid bolus receipt and lactate clearance adjusted for SOFA, EGFR, and MELD

**Fig. 8:**
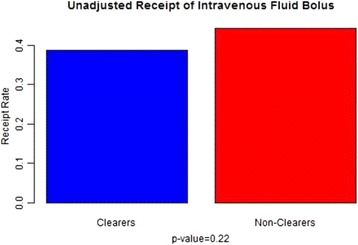
Does not show statistically significant differences in intravenous fluid bolus receipt between the two groups (clearers and nonclearers)

The need for intravenous fluid bolus resuscitation was defined as the receipt of intravenous fluids, including normal saline or lactated ringers administered as a bolus dose of 250 cm^3^ or more, 24 h after the initiation of treatment. The odds of receiving IVF boluses in clearers compared with nonclearers was 0.72 with 95 % confidence interval (0.43–1.22). A test for independence between clearers and nonclearers concluded there was no association with IVF receipt (*p* = 0.22) (Table 2). Sensitivity analysis with multiple logistic regression estimated for the SOFA, EGFR, and MELD adjusted odds ratio of IVF in clearers vs. nonclearers was 0.81 with 95 % confidence interval (0.48–1.39) (*p* = 0.45) (Figs. 7 and 8).

## Discussion

The prognostic potential of plasma lactate and lactate clearance in the management of early sepsis has been repeatedly validated in the critical care literature [2, 9–12, 23–25]. The majority of these investigations have focused on lactate clearance within the initial 6 h of resuscitation, while studies investigating lactate’s utility later in resuscitation consist mostly of small patient populations in surgical and trauma settings [13–17].

Our first finding reveals that nonclearers of lactate (below the median level of lactate clearance) had a higher 30-day mortality rate than clearers. These findings coincide with a 94-patient SICU sepsis study, where Marty et al. measured lactate at time_0_ (*T*), *T*_6_, *T*_12_, and *T*_24_ and showed that the best predictor of death was the *T*_24_ clearance [26]. Similarly, in an 81-patient study, Herwanto et al. investigated the role of 6-, 12-, and 24-h lactate clearance in patients with sepsis and septic shock and showed only the 24-h lactate clearance measurements to be associated with mortality [27].

Regarding vasopressor requirements, we found that patients with lower lactate clearance had a significantly higher requirement for vasopressors than patients with higher lactate clearance after 24 h of sepsis treatment. Vasopressors are used in septic shock to restore and maintain appropriate perfusion pressures to avoid end-organ damage. In our population, clearers after 24–48 h required vasopressors less frequently than nonclearers as their lactate improved. On the contrary, despite continued use of vasopressors to keep adequate perfusion pressures, the low clearance group was unable to improve their lactate levels, while having higher mortality. Thus, in managing patients in late sepsis, restoring adequate perfusion pressure with vasopressors might not be sufficient to reverse all of the causes of hyperlactemia.

Our final finding reveals no significant difference in the requirement for intravenous fluid boluses between the clearers and nonclearers. We believe that this finding is consistent with current treatment approaches calling initially for aggressive fluid resuscitation, followed by vasopressor use only after fluid resuscitation has been established.

New insights into the mechanism for hyperlactemia in sepsis certainly call into question the traditional thinking that hyperlactemia in sepsis and septic shock is only a result of underresuscitation that should be followed immediately with aggressive systemic resuscitation. Instead, the current thinking suggests that after initial resuscitation, the focus should shift more towards infection control and supportive care and less on systemic resuscitation [28–36]. For example, Otto et al. showed that the later phases of sepsis are associated with a significant re-increase in positive blood culture results, especially opportunistic bacteria and fungi [36]. Despite this mechanistic uncertainty, many experts in the field of sepsis continue to support the use of lactate as a marker of early-sepsis recovery [37–39]. However, the role of lactate clearance after initial resuscitation and in late-sepsis management and its potential to guide treatment at these later stages remains unclear [33].

There are inherent limitations in our study worth mentioning. First, our study of 229 patients is limited in size, but this remains larger than any previous studies looking at lactate measurements of septic patients at this specific time period. Consequently, Figs. 3 and 5 show an indirect relationship between SOFA and mortality for clearers at the greatest SOFA values. This anomalous finding can be explained by chance and randomness secondary to few clearers having such high SOFA values. Another potential limitation lies in our study being conducted at a single institution, since variations in treatment protocols among institutions might potentially yield different relationships between lactate and outcomes. Next, there was a statistically significant difference between the clearers and nonclearers with respect to initial lactate values. In fact, we found the clearers to have a higher initial lactate than the nonclearers, which implies that the clearers may have had more severe sepsis (higher initial lactate) on presentation than the nonclearers (lower initial lactate). In prior studies, it has been shown that the initial lactate is directly associated with mortality, but our findings show an indirect association [1–3]. Thus, despite having more severe sepsis, the clearers had less mortality than the nonclearers. This interesting finding can be attributed to the fact that the clearers had a higher late-lactate clearance than the nonclearers. Finally, our results were for nonsurgical patients making extrapolations to surgical patients potentially inaccurate.

There is little debate that early identification and management of septic patients improves outcomes [3, 6, 7]. Our study’s intent was to investigate patients after the initial phase of sepsis treatment to better understand and define late-sepsis patients. The findings in our study shed light on late-sepsis management by looking at late-lactate clearance as a marker to monitor recovery. This study not only illustrates the potential role of lactate clearance as a surrogate for late-sepsis recovery but also suggests that new management strategies should be evaluated for septic patients with a low lactate clearance [40, 41].

## Conclusions

Our study aimed to elucidate whether the utility of lactate clearance early in the resuscitation of septic patients extended further into the course of their care. Our results revealed statistically significant associations between lactate clearance, measured 24–48 h after the initiation of treatment, mortality, and the requirement for vasopressors. Further investigations aimed at early recognition, as well as novel and alternative interventions directed at patients with low lactate clearance after 24–48 h of resuscitation, may reduce sepsis mortality and improve overall outcomes.
